# A quantitative and qualitative evaluation of reports of clinical trials published in six Brazilian dental journals indexed in the Scientific Electronic Library Online (SciELO)

**DOI:** 10.1590/S1678-77572010000200002

**Published:** 2010

**Authors:** Raphael Freitas de SOUZA, Carolina de Andrade Lima CHAVES, Carolina de Andrade Lima CHAVES, Carolina de Andrade Lima CHAVES, Mona NASSER, Zbys FEDOROWICZ

**Affiliations:** 1 DDS, PhD, Professor, Department of Dental Materials and Prosthodontics, Ribeirão Preto Dental School, University of São Paulo, Ribeirão Preto, SP, Brazil, and Visiting Professor, Faculty of Dentistry, McGill University, Montréal, QC, Canada.; 2 DDS, PhD student, Department of Dental Materials and Prosthodontics, Araraquara Dental School, São Paulo State University, Araraquara, SP, Brazil.; 3 DDS, Researcher, Department of Health Information, Institute for Quality and Efficiency in Health Care, Cologne, Germany.; 4 MSc DPH, BDS, LDS RCS (Eng), Director, The Bahrain Branch of the UK Cochrane Centre, The Cochrane Collaboration, Awali, Bahrain.

**Keywords:** Clinical trials as topic, Randomized controlled trials as topic, Evidence-based medicine, Bias, Journalism, dental

## Abstract

Open access publishing is becoming increasingly popular within the biomedical sciences. SciELO, the Scientific Electronic Library Online, is a digital library covering a selected collection of Brazilian scientific journals many of which provide open access to full-text articles. This library includes a number of dental journals some of which may include reports of clinical trials in English, Portuguese and/or Spanish. Thus, SciELO could play an important role as a source of evidence for dental healthcare interventions especially if it yields a sizeable number of high quality reports.

**Objective:**

The aim of this study was to identify reports of clinical trials by handsearching of dental journals that are accessible through SciELO, and to assess the overall quality of these reports.

**Material and methods:**

Electronic versions of six Brazilian dental Journals indexed in SciELO were handsearched at www.scielo.br in September 2008. Reports of clinical trials were identified and classified as controlled clinical trials (CCTs - prospective, experimental studies comparing 2 or more healthcare interventions in human beings) or randomized controlled trials (RCTs - a random allocation method is clearly reported), according to Cochrane eligibility criteria. Criteria to assess methodological quality included: method of randomization, concealment of treatment allocation, blinded outcome assessment, handling of withdrawals and losses and whether an intention-totreat analysis had been carried out.

**Results:**

The search retrieved 33 CCTs and 43 RCTs. A majority of the reports provided no description of either the method of randomization (75.3%) or concealment of the allocation sequence (84.2%). Participants and outcome assessors were reported as blinded in only 31.2% of the reports. Withdrawals and losses were only clearly described in 6.5% of the reports and none mentioned an intention-totreat analysis or any similar procedure.

**Conclusions:**

The results of this study indicate that a substantial number of reports of trials and systematic reviews are available in the dental journals listed in SciELO, and that these could provide valuable evidence for clinical decision making. However, it is clear that the quality of a number of these reports is of some concern and that improvement in the conduct and reporting of these trials could be achieved if authors adhered to internationally accepted guidelines, e.g. the CONSORT statement.

## INTRODUCTION

There has been an increased trend over the last decade towards open access publishing of health science literature^8^, the main goals of which are the improvement of scholarly interaction, the creation of options for sharing knowledge without cost and with a perception that this will lead to a reduction in the barriers to the growth of science. In practical terms, published articles should be available on the internet and should permit users to read, print and distribute the documents without cost^9^. Despite the obvious advantages for consumers of healthcare science, the increasing popularity of open access publishing has had an impact on healthcare researchers. A study of the perceptions of American medical researchers to open access publishing found that the concept of free access was a significant influence in decision making about where to publish research. Free access was the main reason for considering open access journals, in addition to a belief that it would increase the visibility of research findings^19^.

The Scientific Electronic Library Online (SciELO) has previously been highlighted as a successful example of open access publishing^6^. It houses a collection of dental journals, which receive manuscripts in English, Portuguese and/or Spanish, many of which might include clinical trials, systematic reviews which could be an important source of evidence for effectiveness of interventions in dentistry. This perception is in keeping with similar findings from other regions which showed that many healthcare journals are a rich resource of high quality clinical trials, irrespective of their lack of indexing in the larger international databases. A study in which ten German dental journals were handsearched for randomized clinical trials (RCTs) retrieved more than 200 reports, out of which 43.8% were not available in MEDLINE^18^. A number of Iranian medical journals have also been shown to contain a considerable number of trials which are not available through MEDLINE or EMBASE^16,17^. Similar results have been found with Polish journals where 40% of the reports of RCTs were not retrievable by means of a search on MEDLINE^1^.Thus it is conceivable that SciELO dental journals contain a substantial number of reports of clinical trials which are not widely accessible but which could be an important source of both evidence for effectiveness of healthcare interventions and a potential source of eligible studies for systematic reviews.

Regardless of the number of reports of trials in SciELO it is essential that they are robust in methodological quality and that reasonable attempts have been made by investigators to minimize the risk of systematic bias. Control of bias in clinical trials is directly related to several important procedural aspects of trial conduction, i.e. random and concealed allocation to intervention and control and the effective blinding of participants, investigators and outcomes assessors^14^. A comprehensive accounting for losses or withdrawals is a prerequisite in the control of bias and any losses to follow up or withdrawals should be adequately described in the reports^13^.

The role of SciELO as a source of high-level evidence for the effectiveness of oral healthcare interventions can best be illustrated through a comprehensive examination of the quality of reports of clinical trials published in journals accessible through this database. This study sought to identify and assess the risk of bias in reports of clinical trials published in Brazilian dental journals that are accessible through SciELO.

## MATERIALS AND METHODS

A pilot search of the SciELO database, via www.scielo.br, was conducted in February and subsequently updated in September of 2008. Journals listed under the heading “health sciences” were assessed for eligibility based on two principal criteria; the selected journal focused on general dentistry or a dental specialty or on related topics, and was a Brazilian journal indexed in the Brazilian section of SciELO. There were no language restrictions and both active and inactive titles were eligible for inclusion.

Each issue of the included journal was handsearched via the SciELO home page, and although the handsearching was conducted on electronic copies rather than print issues the methods used were as described in the Cochrane Oral Health Review Group Journal studies were considered eligible based on four criteria: (I) healthcare treatment or interventions were compared, in human beings; (II) prospective and experimental study; (III) two or more treatments or interventions were compared to one another or to a control (inactive) group; (IV) assignment of participants, parts of the body or clusters to treatments or interventions was intended to be random. In other words, a report was not eligible for inclusion if participants were explicitly assigned to interventions using some method other than randomization or quasi-randomization. If eligible, these reports were further classified according to the method used for random assignment, according to two categories:

1. RCT: the report clearly stated that random allocation was used, or described a procedure such as the use of computer-generated codes or random number tables for defining assignment;

2. Controlled clinical trial (CCT): the report did not cite the use of random allocation or true random methods. This classification was also employed for articles which cited the use of quasirandom methods, such as alternate allocation or use of social security numbers.

The methodological quality of the trial reports was assessed based on the criterion grading system described in the Cochrane Handbook for Systematic Reviews of Interventions 5.0.0^12^ (Figure 1). All search and quality assessment procedures were carried out independently by two researchers (CALC and RFS). Disagreements in assessment were discussed and resolved through consensus and if necessary through consultation with a third party, which was either (MN or ZF).

**Figure 1:** Criteria for quality assessment

**Criterion**	**Classification**
Randomization	(A) adequate - include any one of the following methods of sequence generation: computer generated or table of random numbers, drawing of lots, coin-toss, shuffling cards or throw of a dice; (B) unclear; (C) inadequate - sequence generation using any of the following: case record number, date of birth or alternate numbers.
Concealment of allocation	(A) adequate - either central sequence generation or sequentially numbered sealed opaque envelopes; (B) unclear; (C) inadequate - open allocation sequence and the participants and trialists could foresee the upcoming assignment.
Blinding	(a) blinding of participants (yes/no/unclear/not applicable); (b) blinding of caregiver (yes/no/unclear/not applicable); (c) blinding of outcome assessor (yes/no/unclear/not applicable); (d) blinding of data analyst (yes/no/unclear/not applicable).
Handling of withdrawals and losses	(A) yes (there was a clear description given of the difference between the two groups of losses to follow up); (B) unclear; (C) no.
Intention-to-treat(ITT) analysis	(A) yes (ITT was mentioned among the analyses); (B) unclear; (C) No (per-protocol analysis); (D) Not applicable (there was no withdrawal or loss at follow-up).

As this was a descriptive study, data for quality assessment was presented only as frequency counts.

## RESULTS

The initial search retrieved six titles for eligible journals (Figure 2). The titles A, B and C were for three independent journals, whereas the titles D and E were not being published anymore. Both were continued by the title F. A considerable number of reports of clinical trials and a few systematic reviews were identified in the journals which were searched.

**Figure 2:** Characteristics of the Brazilian Oral Health journals in SciELO and classification of reports

**Journals**	**Characteristics**			**Classification**		
	**Frequency**	** Issues**	**Subject**	**CCT**	**RCT**	** Total**
(A) Revista Dental Press de Ortodontia e Ortopedia Facial * (Dental Press* * Journal of Orthodontics and Facial* * Orthopedics)*	Bimonthly	20	Orthodontics	2	2	4
(B) Brazilian Dental Journal	Quarterly	21	General	8	8	16
(C) Journal of Applied Oral Science	Bimonthly	27	General	9	15	24
		+ 2 Special Issues				
(D) Revista de Odontologia da Universidade de São Paulo *(University * *of São Paulo Dental Journal)*	Quarterly	11	General	4	0	4
		+ 1 Supplement				
(E) Pesquisa Odontológica Brasileira *(Brazilian Oral Research)*	Quarterly	16	General	7	6	13
		+ 1 Supplement				
(F) Brazilian Oral Research	Quarterly	18	General	3	12	15
**Total**				**33**	**43**	** 76**

**Figure 3:**
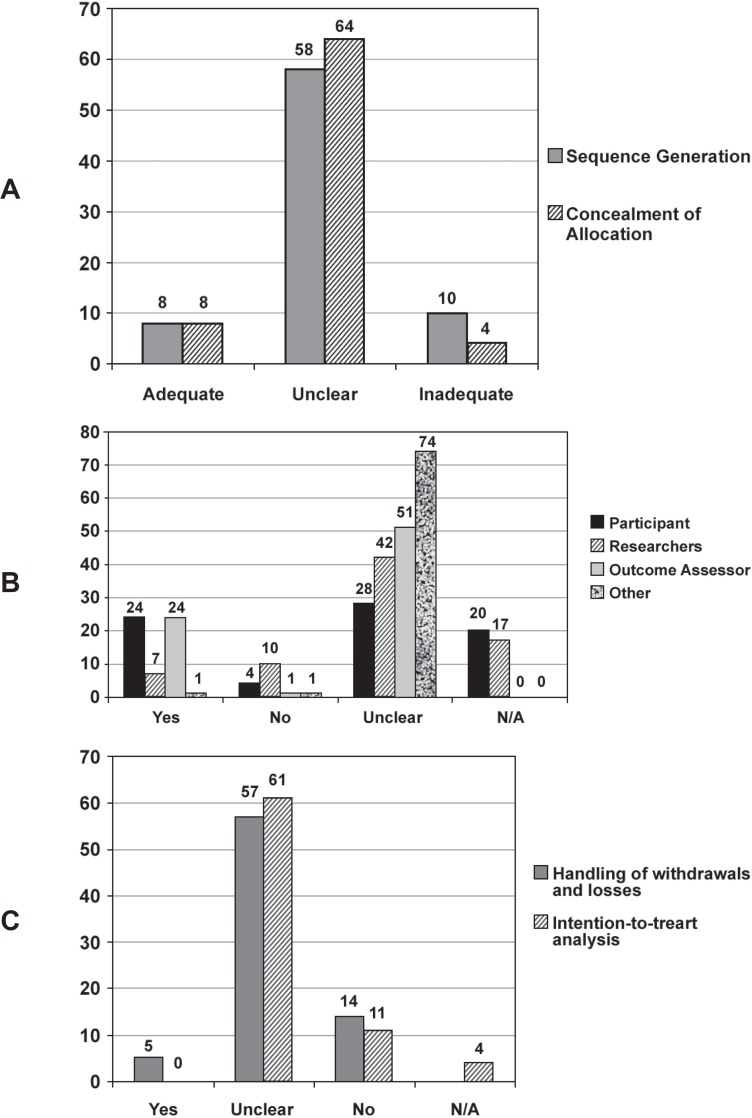
Results for methodological quality assessment. (A) Randomization and concealment of allocation, (B) Blinding, and (C) Handling of withdrawals and losses and intention-to-treat analysis. N/A: not applicable

Quality assessment of the reports illustrated that 75.3% of the reports did not explain how participants were randomized, and 84.2% did not indicate the methods used to conceal the allocation sequence (Figure 3A). Of the included reports, 13.2% and 5.3% respectively, confirmed inadequate methods for the randomization and concealment of allocation criteria.

The most frequently used method for control of bias was blinding of participants or assessment of outcomes, which were both described in 31.6% of the reports. Blinding of caregivers was much less frequent (9.2%); whereas data analysts were seldom described as blinded (unclear for 97.4%) (Figure 3B). Despite this higher frequency of reports with adequate blinding, most of the studies were either underreported or appeared to be of low methodological quality.

Handling of withdrawals and losses was unclear for a large proportion of the reports (75.0%), and 18.4% of the reports provided inadequate descriptions (Figure. 3C). None of the reports of trials made any mention of an intention-to-treat analysis, although it was clearly necessary in more than 15% of them.

## DISCUSSION

This study confirmed the availability of a substantial number of reports of clinical trials and systematic reviews in Brazilian dental journals listed in the SciELO database. However, although the findings reinforce the relevance of those journals as a source of evidence for the effectiveness of oral healthcare interventions there are several important implications to this current study.

Firstly, SciELO should be considered a useful resource for clinical decision making in dentistry in much the same way as a number of other regional databases, in both medicine^1,16^ and oral health^18^. Those databases, as with SciELO, contain a considerable number of reports which are not currently indexed in MEDLINE or EMBASE, and their relevance for regional settings would justify their use. Secondly, additional benefits in strengthening the evidence base for effectiveness of oral healthcare interventions would be accrued through their inclusion in search strategies for systematic reviews. It is recognised that searches which are restricted to international databases such as MEDLINE may result in a higher risk of bias than more comprehensive approaches, depending on the nature and direction of the results^7^. Future studies might attempt to develop and test search filters for SciELO, which could assist in extracting previously inaccessible reports of trials.

Quality assessment of the trials identified for evaluation revealed a huge proportion of reports with methodological deficiencies. Procedures critical for the control of selection bias^14^, such as those associated with randomization and allocation concealment were infrequently described in the reports and which in some instances highlighted the use of an inadequate procedure. Inadequate randomization can result in differences among groups at baseline and thus differences in outcomes may be incorrectly attributed to confounding variables instead of the tested interventions^5^. Open allocation can lead to bias by providing conditions for excluding certain participants from one intervention group or the other^14^. Open allocation may facilitate the allocation of a more complex case to the placebo rather than the active intervention arm of a trial.

Somewhat surprisingly, blinding was more frequently employed, although most reports were unclear about who was blinded and precisely how and when this was achieved. Several studies had one or more interventions or situations where blinding would not be applicable or feasible, i.e. it would not be possible to blind participants to conventional complete dentures or implantretained overdentures in a trial which included edentulous patients^15^. Not one single study reported blinding of data analysts and seldom were caregivers reported as blinded. Several reports cited the use of double-blinding, but did not indicate who was blinded and at which stage of the study. Blinding is essential for the control of performance and detection bias and should be viewed as being quite distinct from allocation concealment, and even though both aim to control bias they are employed at different stages of a trial. Performance bias is minimized by adequate blinding and refers to possible systematic differences in the care provided for participants of each group, other than those planned a priori. The blinding of outcome assessors, if feasible, can also reduce detection bias, attributed to systematic differences between groups during outcome assessment^12^.

Attrition bias which was a further concern in our findings refers to systematic differences between groups due to the loss of participants during a study^14^. Despite its relevance, only 5 reports adequately cited how many enrolled participants completed the studies. Not a single study cited the use of intention-to-treat analysis, which was quite surprising in view of the generally inadequate description of withdrawals and losses. Missing data is a common theme even in studies published in leading medical journals^10^, it is tempting therefore to assume that several of the reports assessed in this study did not conduct an intention to-treat-analysis if appropriate, more especially as in many instances any reference to withdrawals and losses was omitted.

An important consideration of this study is that a distinction should be made between the quality of reporting and the methodological quality of the trials and the only way to disentangle these would be through direct contact with the investigators in the trials. However, excessively positive responses can be expected when authors are asked about control of bias and this is not infrequently accompanied by over optimism^11^.

An earlier study evaluating the quality of clinical trials conducted in juvenile idiopathic arthritis noted that there was a significant improvement in the quality of reports after 1996^4^. According to the authors, this improvement was most probably due to the publication of the CONSORT statement and to the establishment of international networks for the conduct of high-quality trials in children with rheumatic diseases^2^. Thus, one might expect significant improvements in trial quality if both authors and editors of Brazilian dental journals adhered to the guidelines, such as those expressed by the Cochrane Collaboration or the CONSORT statement. In addition it would be helpful if, authors of trial protocols consider the possibilities of working in groups which include at least one methodologist. A more ambitious approach for the improvement of trial quality would be the establishment of networks for discussing and planning clinical experimental studies in dentistry.

## CONCLUSIONS

A substantial number of reports of clinical trials are available in dental journals accessible through the SciELO database. Although these trials can provide valuable evidence for clinical decision making, the present assessment showed that the quality of many of these reports is a concern, and that future improvements in trial conduction are likely to be driven by authors adhering more closely to internationally accepted guidelines.
